# Survey data of adolescents’ sexual and reproductive health in selected local governments in southeast Nigeria

**DOI:** 10.1038/s41597-020-00783-w

**Published:** 2020-12-17

**Authors:** Chinyere Ojiugo Mbachu, Ifunanya Clara Agu, Obinna Onwujekwe

**Affiliations:** 1 Health Policy Research Group, University of Nigeria Nsukka, Enugu, Nigeria; 2 Department of Community Medicine, University of Nigeria Nsukka, Enugu, Nigeria; 3 Department of Health Administration and Management, University of Nigeria Nsukka, Enugu, Nigeria

**Keywords:** Medical research, Social sciences

## Abstract

A cross-sectional survey of adolescents and heads of households was done in six urban and rural local government areas in Ebonyi state, Nigeria in August 2018. Modified cluster sampling technique was used to select households from which eligible adolescent boys and girls were recruited. This data article describes two datasets that, for the first time, expansively describe adolescents’ sexual and reproductive behaviors in Nigeria. The datasets include variables on adolescents’ demographic and socioeconomic characteristics; family relationships; sexual behaviors; awareness and use of contraceptives; access to sexual and reproductive health information and services; gender norms and ideology about adolescent sexuality; and potential strategies for reducing unwanted teenage pregnancies and unsafe abortions. This dataset would be useful to public health researchers and social scientists investigating drivers of adolescent sexual and reproductive behaviour, as well as programme managers seeking potential strategies for improving adolescent health outcomes. The datasets also provide a template that could be replicated for national or regional surveys on adolescent sexual and reproductive behaviours.

## Abstract (summary)

**Table:** 

Measurement(s)	Sex Behavior • reproductive behavior
Technology Type(s)	Survey
Factor Type(s)	local government area • age • gender
Sample Characteristic - Organism	Homo sapiens
Sample Characteristic - Location	Ebonyi State

Machine-accessible metadata file describing the reported data: 10.6084/m9.figshare.13341953

## Background & Summary

Efforts to prioritize adolescent health in Nigeria have led to the development of policies^1,2^, and the inclusion of ‘adolescent’ in reproductive health terminology. Nevertheless, access to sexual and reproductive health (SRH) information and services for adolescents is low/suboptimal in Nigeria^3–6^. In order to design and successfully implement delivery strategies, we need to understand the nature and determinants of sexual and reproductive behaviors among adolescents. Although adolescents are included in national surveys in Nigeria, and results are reported for different age categories (including 15–19 age group), there is no dataset that expansively describes adolescent sexual and reproductive behaviors. A situation analysis was undertaken as part of an implementation research project to address the SRH needs of adolescents in Ebonyi state. The situation analysis adopted a mixed-method approach of data collection, but this data article describes the dataset from the quantitative survey.

A cross-sectional survey of adolescents and households was done in six communities in Ebonyi state. Modified cluster sampling technique was used to select households from which heads of households and eligible adolescents were interviewed. Data was collected by trained research assistants using structured pre-validated questionnaires. The objectives of the survey were to determine at baseline, i) the sexual (and other lifestyle risky) behaviors of adolescents; ii) their knowledge and use of contraceptives; iii) their access to sexual and reproductive health information and services; iv) the relationship between adolescents’ sexual/reproductive behavior and their socio-demographic characteristics and socioeconomic status; and v) perception of interventions for addressing the sexual and reproductive health needs of adolescents. A research paper on parent communication of SRH matters with adolescents has been published in BMC Public Health^7^.

The datasets will be particularly useful to researchers investigating demographic, socioeconomic and other correlates of adolescent sexual and reproductive behaviour; social scientists seeking to understand gender norms and ideologies that drive adolescent SRH; and programme managers and policymakers seeking strategies to improve access to SRH information and services for adolescents. The dataset also provides a complete template that could be replicated for large scale (national or regional) surveys on adolescent sexual and reproductive behaviors.

## Methods

### Study area

This survey was conducted in Ebonyi State, south-East Nigeria. Based on the 2006 census and an annual growth rate of 3.4%, Ebonyi state was estimated to have a 2017 population of 6,268,003 inhabitants that are spread across 5,935 square kilometres. Ebonyi state has 13 local government areas (LGAs), out of which 6 were purposively selected for this study. The study LGAs were selected to ensure geographic and geopolitical spread in terms of place of residence (urban and rural) and the senatorial zones. In each geopolitical zone we aimed to select LGAs that that had been prioritized by the State government for adolescent sexual and reproductive health interventions.

### Ethical considerations

Ethical approval to undertake the research was obtained from the Health Research Ethics Committee of University of Nigeria Teaching Hospital with reference number NHREC/05/01/2008B-FWA00002458-IRB00002323. Approval was also obtained from the ethics committee of Ebonyi State Ministry of Health. Data sharing and publication were discussed during the project ethics review, and consent for data sharing and publication were sought from participants during data collection. These approvals were secured prior to entry into the study sites. The principles of ethical conduct of research involving humans were observed including respect for autonomy expressed through voluntary informed consent, beneficence expressed through favorable balance of benefits and risks, justice expressed through fair inclusion, and privacy of information ensured by anonymized collection and use of data. The dataset is anonymized by non-inclusion of direct identifiers. Although we retained indirect identifiers such as LGA and community codes in the dataset, we do not describe these codes in the codebooks.

In addition to securing ethical approval, the research team held a meeting with key stakeholders in adolescent health in the State to introduce the research project and seek their buy-in. Advocacy visits were also made to key community leaders to obtain permission to enter their communities and interview adolescents.

Informed consent/assent was sought from all eligible participants. Participants were informed of the purpose of the research, rights of participants and measures that will be taken by researchers to protect them and their data. Consent was obtained from the head of household or an adult representative who provided information for the household questionnaire. Parental/guardian’s informed consent was sought for all eligible adolescents aged 13 to 17 years within a household. This was followed by individual assent to participate from these adolescents. Adolescents that were 18 years of age and mature minors aged 15–17 years gave consent for themselves. For this study, a mature minor was defined as an adolescent who is no longer under the care of a parent or guardian.

The head of household or adult representative consented at the same time to being interviewed about the household; and for household members aged 13–17 years to be interviewed. Documentation of informed consent was through signature or thumb print, as appropriate, and paper copies of consent forms are being stored in a locked, fire proof cabinet.

Household members who had cognitive disabilities that preclude them from consenting or giving assent were excluded from participating. Cognitive and other disabilities were assessed on a case-by-case basis. Participants aged 13–14 years who were no longer under the care of a parent/guardian were not eligible to give consent for themselves.

### Study design

A cross-sectional study design was used. Eligible households and adolescents were surveyed using pre-tested interviewer-administered questionnaires namely, head of household questionnaire (HHQ) and adolescent SRH questionnaire. The head of household questionnaire was administered on the heads of selected households or their representatives. The adolescent questionnaire was administered to eligible adolescents in selected households.

### Study population

The study population consisted of heads of selected households and unmarried adolescents aged 13–18. The age of sexual debut in Ebonyi State ranges from 13–15 years. The study targeted unmarried adolescents aged 13–18 years following recommendations by key stakeholders in Ebonyi State that the population of interest are adolescents of secondary-school age (which in the State typically ranges from 13–18). Only households that had adolescents within 13 to 18 years of age were included in the household survey. Households whose heads were absent at the time of the survey and those whose heads are less than 18 years old were excluded from the survey.

Adolescents aged 13 to 18 who are unmarried and living in selected households were included in the adolescent SRH survey. The survey excluded adolescents who were visitors or guests in selected households, married adolescents, those who were in a form of stable co-habiting relationship (even if their partner was away during the survey), and those who had hearing, sight or mental impairment.

### Sample size and Sampling technique

In order to achieve 5% precision at 95% confidence interval for population >100,000 a minimum sample size of 400 was determined from Glenn’s table of sample sizes that would be necessary for given combinations of precision, confidence level and variability for different population sizes^8^. This was doubled to enable sub-group (urban-rural) analysis of data, and further increased to 1,100 for robustness and to account for incomplete responses or errors in questionnaires.

Modified cluster sampling technique was used to select households from which eligible adolescents were recruited. One cluster (defined as an autonomous village) was selected from each community. An autonomous village is defined as a community which is governed by an appointed or elected traditional ruler. Within each cluster, the nearest public facility (primary health centre, school church or town hall) from the main entrance was identified as the starting point from which households were consecutively recruited. The first household in each cluster was selected through a random walk from the starting point; subsequently the nearest households were selected consecutively until the desired sample size was reached. All eligible adolescents in selected households were invited to participate in the survey. All visitors or guests were excluded. One repeat visit was made to each household to recruit adolescents who were absent during the first visit.

### Data collection

Data was collected using two structured questionnaires, head of household and adolescent questionnaire. The ensuing paragraphs provide detailed descriptions of the processes of adaptation and validation of the questionnaires, recruitment and training of research assistants for the survey and administration of survey instruments.

#### Adaptation and validation of adolescent questionnaires

The adolescent questionnaire was adapted from WHO illustrative questionnaire for interview-surveys with young people^9^. This questionnaire is suitable for unmarried teenagers and young people who have not entered stable co-habiting relationships. It is equally suitable for males and females, for those who are attending school and those who are not, and for sexually exposed and unexposed people. This illustrative questionnaire which was designed to document knowledge, beliefs, behaviour and outcomes in the domain of sexual and reproductive health, is an effective tool for assessing the needs and problems of young people prior to an intervention. It collects a wide range of information on adolescents’ background characteristics; sexual conduct; sexual ideology; protective or risk behaviour; knowledge and use of condoms; knowledge, perception and utilization of sexual and reproductive health information and services; and sexual and reproductive health outcomes.

The questionnaire was adapted by rephrasing some questions, re-ordering some sections, adding more options to questions, adding new section of questions, and deleting some questions altogether. The adapted questionnaire had 7 sections namely: demographic characteristics; socioeconomic characteristics and family relationship; information on sexual and reproductive health; sexual conduct; awareness and use of contraceptives; use of sexual and reproductive health services; ideology and gender norms; and potential interventions for improving adolescent SRH (particularly for reducing unwanted teenage pregnancy and abortions).

The questionnaire was reviewed by a team of experts to ensure that the subject area was sufficiently covered and that only relevant items were included. It was then pre-tested on 24 adolescents that were purposively selected to equally represent place of residence (urban and rural), gender (male and female), and schooling (attending school and not attending school). We also ensured that all the ages of interest were represented. The purpose of the pre-testing was to ensure our theoretical constructs were well operationalized in the instrument and groups of adolescents did not understand them differently.

#### Adaptation and validation of household questionnaires

The household questionnaire was adapted from a questionnaire developed by researchers from Health Policy Research Group University of Nigeria and Nuffield Centre for International Health & Development University of Leeds, for use in a community-based household survey. This questionnaire was recently used by Health Policy Research Group, University of Nigeria, during a community-based survey of households to collect information on background characteristics and expenditure patterns of selected households. The questionnaire was adapted to this study by inclusion of questions on household listing of adolescents and a section on potential interventions for improving adolescent SRH (particularly for reducing unwanted teenage pregnancy and abortions).

The process of validation of the questionnaire is similar to that of adolescent questionnaire. It was reviewed by a team of experts and pre-tested on 4 heads of households that were purposively selected to equally represent place of residence (urban and rural) and gender (male and female).

#### Recruitment and training of research assistants

Fifty-four research assistants were recruited and trained for 5 days to enable them administer the survey instruments properly. Established researchers in the State were approached and asked to share the names and telephone or email contacts of young people they had previously trained to assist with collecting quantitative data. A list of potential enumerators was compiled and they were invited for a screening interview. The screening interview was conducted by a team of researchers from Health Policy Research Group. A uniform set of criteria consisting of objectively structured questions to assess intellectual and technology proficiency was developed and used to examine each potential enumerator. Each candidate was scored minimum of 0 (for no proficiency) and maximum of 5 (for perfect proficiency) in each question. Candidates’ scores were totaled and those who scored less than half of the maximum were screened out. Those who scored 50% and above were further screened for language proficiency and only those who could communicate (speak and understand) in the local language as well as in English were invited for the training.

The training of research assistants was facilitated by the principal investigator, six research fellows, and two IT/data software consultants. The objectives of the training were to get the research assistants to: become familiar with the overall study; become knowledgeable about the objectives of the survey and the study population; fully understand the constructs in the survey instruments; full understand the processes of data collection and management; and become proficient in administering the survey instruments. The training consisted of didactic and interactive plenary sessions, and parallel group work sessions. Role plays were introduced on the third day and research assistants worked in parallel groups, alternating roles as interviewers, recorders and respondents. The role plays were supervised by research fellows who observed, provided feedback and made corrections where necessary.

The level of knowledge of research assistants was assessed objectively, using structured questions with multiple choice options, at the beginning of the training and on the fourth day of the training. Their proficiency in administering the survey instruments was evaluated through actual field practice on the fourth day of training.

#### Administration of questionnaires

Paper and electronic copies of questionnaires were administered to eligible heads of households and adolescents as appropriate. Electronic copies of the questionnaires were built on SurveyCTO Collect v2.70 and uploaded to android tablets for data collection. Each respondent was surveyed by a pair of research assistants consisting of an interviewer (who asks the questions and enters responses in the electronic questionnaire) and a recorder (who records responses on the paper questionnaire). Data was collected daily from Monday to Saturday ideally, and from 7am to 6 pm ideally. On rare occasions, research assistants were asked to return after 6 pm and on Sundays to be able to meet with heads of households and obtain permission to interview adolescents in such households. Data collection lasted for a period of ten days.

#### Estimation of household wealth index

The household wealth index is a composite of household consumption pattern that places households on a continuous scale of relative wealth. Total household consumption can be calculated by adding total food and non-food expenditure (m2q06_totat & totttt). Per capita household consumption will then be calculated by dividing total household consumption by number of children in the household (m1q05_nochildren). The per capita household consumption can be used to categorize households into wealth index groups such as quintiles Q1 to Q5, where Q1 refers to poorest households and Q5 refers to richest households.

The sample weights were calculated per community using formula, W = n_(a-f)_/N

(Where n = number of adolescents from community a to f; N = total number of adolescents in the survey)

A total of six (6) sample weights were calculated.

The same sample weight was applied to adolescents in the same community.

## Data Records

The datasets have been uploaded to UK Data Service in Excel CSV and Stata software formats which can easily be imported into a variety of software programs^10^. Dataset 1 contains information elicited from heads of households where adolescents reside. It has over 350 variables structured into background information and 3 modules namely, Module 1: Socio demographic characteristics; Module 2: Socio economic status of household (including household expenditure on food and non-food items); and Module 3: Potential interventions for improving adolescent SRH (particularly for reducing teenage pregnancy and abortions)

Dataset 2 contains information elicited from adolescents. It has over 300 variables structured into background information and 8 modules namely, Module 1: Socio demographic characteristics; Module 2: Socio economic characteristics, family relationship, and lifestyle risks; Module 3: Access to information on sexual and reproductive health; Module 4: Sexual relationships/behaviour; Module 5: Awareness and use of contraceptives; Module 6: Access to sexual and reproductive health services; Module 7: Ideology and gender norms; and Module 8: Potential interventions for improving adolescent SRH (particularly for reducing teenage pregnancy and abortions).

A full description of the variables in the datasets is available in a data dictionary in the repository.

## Technical Validation

In addition to rigorous training of research assistants and use of paper-back up questionnaire, other measures were taken to ensure that good quality of data was collected from respondents, and they include: (1) two layers of supervision of field work activity; (2) concurrent viewing of data as field work activity was going on; (3) individual matching of information on completed paper-questionnaire with corresponding electronic-questionnaire, before and after uploading data to the server.

A team of research assistants was made up of nine people comprising of four pairs of enumerators and one supervisor. The supervisors moved with their teams on a daily basis. They were responsible for introducing enumerators to an eligible household and obtaining relevant informed consent from the head of household. They were also responsible for retrieving all tablets and completed paper questionnaires from enumerators on a daily basis, conducting individual matching of paper and electronic data for each questionnaire, and uploading correctly matched questionnaires to the server. The second layer of supervision was performed by zonal coordinators.

Three zonal coordinators were selected from the team of research fellows who participated in training the research assistants. Each zone consisted of two teams of research assistants (that is eight pairs of enumerators and two supervisors), and the zonal coordinator was responsible for overseeing the activities of these teams. The zonal coordinators visited the study sites on at least three different occasions during data collection, one of which was the first day of field work, to ensure that the survey protocol for data collection was strictly being adhered to. They were also responsible for undertaking concurrent data viewing, as questionnaires were being uploaded to the server, to detect irregularities in data entry and inconsistencies in responses.

On-going analysis and sorting of electronic data was performed by the data consultant on a daily basis. Observed inconsistencies and errors were immediately flagged up and enumerators were notified.

The last stage of data quality management was done at the end of data collection. The complete data set was first downloaded using Excel software, and correction of errors that were identified and rectified during concurrent data viewing was done. Individual matching of information on completed paper-questionnaire with respondent’s data on the Excel spreadsheet questionnaire was repeated for all questionnaires. In order to ensure that individual data in the paper questionnaire corresponds with electronic data, researchers checked the response for each question at this last stage of data cleaning and inconsistent or poorly matched questionnaires were deleted from the data set.

## Usage Notes

These data can be used by public health researchers to investigate demographic, socioeconomic and other correlates of adolescent sexual and reproductive behaviour in Ebonyi, state and elsewhere. Social scientists seeking to understand gender norms and ideologies that drive adolescent SRH will also find these data useful. Programme managers, implementers and policymakers who are seeking strategies to improve access to sexual and reproductive health information and services for adolescents will find these data useful. Funders and designers of national and regional surveys may also find the dataset a useful resource because it provides a complete template for capturing adolescent sexual and reproductive behaviors at scale.

Although we have made considerable efforts to ensure data is of good quality, those who wish to use the datasets need to be mindful of the following limitations of the data in reporting their findings. The adolescent questionnaire was administered to only unmarried adolescents aged 13–18 years who were at home during the time of the survey. This systematically excludes married adolescents, adolescents aged 10–12 and 19, those who were not at home during the survey (including those who had gone to skill acquisition, apprenticeship or trade centers). The exclusion of these categories/groups of adolescents limit the scope of our dataset and generalizability of findings from data analysis to all adolescents. Another limitation of the data arises from the purposive selection of study sites to reflect prioritization by the State government for adolescent health intervention, and this could mean areas with the poorest health indices or areas that have received relatively more attention from government in terms of resources and interventions. This study may be comparable to other surveys that applied (adopted or adapted) the WHO instrument. However, in making any comparisons, researchers should be cautious of the specific characteristics of our study population which comprised only unmarried adolescent boys and girls aged 13–18 years. If any errors or omissions are found, users of the datasets are encouraged to contact us directly by emailing the corresponding author.

## Data Availability

The data were generated using SurveyCTO Collect v2.70, and downloaded using Microsoft Excel 2013. The datasets were then saved in CSV format and imported to Stata software. All codes that were used to clean the data (drop multiple columns, check missing data and remove duplicates) have been uploaded to the repository^10^.
